# Pyroptotic cell death: an emerging therapeutic opportunity for radiotherapy

**DOI:** 10.1038/s41420-024-01802-0

**Published:** 2024-01-16

**Authors:** Hongbin Li, Tiantian Yang, Jialin Zhang, Kai Xue, Xiaoli Ma, Boyi Yu, Xiaodong Jin

**Affiliations:** 1 School of Life Science and Engineering, Lanzhou University of Technology, Lanzhou, 730050 China; 2 Institute of Modern Physics, Chinese Academy of Sciences, Lanzhou, 730030 China

**Keywords:** Radiotherapy, Tumour immunology

## Abstract

Pyroptotic cell death, an inflammatory form of programmed cell death (PCD), is emerging as a potential therapeutic opportunity for radiotherapy (RT). RT is commonly used for cancer treatment, but its effectiveness can be limited by tumor resistance and adverse effects on healthy tissues. Pyroptosis, characterized by cell swelling, membrane rupture, and release of pro-inflammatory cytokines, has been shown to enhance the immune response against cancer cells. By inducing pyroptotic cell death in tumor cells, RT has the potential to enhance treatment outcomes by stimulating anti-tumor immune responses and improving the overall efficacy of RT. Furthermore, the release of danger signals from pyroptotic cells can promote the recruitment and activation of immune cells, leading to a systemic immune response that may target distant metastases. Although further research is needed to fully understand the mechanisms and optimize the use of pyroptotic cell death in RT, it holds promise as a novel therapeutic strategy for improving cancer treatment outcomes. This review aims to synthesize recent research on the regulatory mechanisms underlying radiation-induced pyroptosis and to elucidate the potential significance of this process in RT. The insights gained from this analysis may inform strategies to enhance the efficacy of RT for tumors.

## Facts

- The effect of radiotherapy is not limited to local control of the lesion, and its immunomodulatory effect is also worth utilizing.
- Radiation can induce immunogenic cell death (ICD).
- Pyroptosis is an ICD that could be triggered by radiation and has been found to sensitize cancer cells to radiation.
- The mechanism by which pyroptosis initiates an antitumor immune response after radiotherapy is not clear.
- The positive role of radiation-induced pyroptosis in combination therapy with radiotherapy and immunotherapy is anticipated.

## Open questions

- What is the correlation between cell death in tumors induced by radiotherapy and the subsequent immune response against tumor growth?
- How does radiation induce pyroptosis?
- What strategies can be employed to specifically target radiation-induced pyroptosis in order to enhance the efficacy of tumor therapy?
- Can pyroptosis-modulating drugs serve as sensitizers in clinical radiotherapy?

## Introduction

Radiotherapy (RT) is the primary regimen for clinical cancer treatment, and it is estimated that about 50% of patients will undergo RT during their treatment. RT is significant for improving local tumor control, prolonging overall survival time, and improving patients’ quality of life. However, radioresistance, which may lead to tumor recurrence and metastasis, remains the main cause of RT failure [1]. Therefore, the focus of radiation oncology has been on improving the effectiveness of RT and reducing adverse reactions significantly. The traditional view has been that RT plays a direct antitumor role by inducing tumor cell death through DNA damage. Still, the recent views are that the antitumor function of RT depends on the immune system more [2]. For example, in the clinical treatment of metastases, it has been found that local irradiation of a specific tumor site can cause the tumor volume of non-irradiated fields to shrink significantly or even disappear, a phenomenon called the distal effect (abscopal effect) [3]. Although the mechanisms behind it are not fully understood, the “in situ” vaccine effect of RT and the modification of the tumor immune microenvironment by RT are thought to be the main reasons for the abscopal effect. Some findings have helped to shed light on how combining RT with immunotherapy would facilitate this effect [4].

According to the degree of immunogenicity, cell death can be divided into non-immunogenic death (such as apoptosis, which plays an important role in maintaining the homeostasis of body development) and immunogenic cell death (ICD) (such as pyroptosis, necrotic apoptosis, ferroptosis, which can cause a strong immune response). Pyroptosis is an immunogenic death caused by inflammasomes, in which cells continuously expand until the cell membrane bursts, releasing cell contents and triggering a robust inflammatory response. Pyroptosis is mediated by the inflammatory caspase and GSDMs protein family. In short, activated caspase cleaves the GSDMs protein, releasing its N-terminal domain, which binds to membrane lipids and perforates the cell membrane, leading to changes in osmotic pressure and swelling until the cell membrane bursts. As a recently discovered form of cell death, pyroptosis has garnered escalating interest within the realm of cancer RT. This is due to its dual capability of directly inducing tumor cell death and stimulating the immune system to eradicate any lingering cancer cells subsequent to RT, thereby potentially augmenting the therapeutic efficacy. It has been shown that after radiation exposure, tumor cells release damage-associated molecular patterns (DAMPs) that can cause pyroptosis, which serves a critical role in ionizing radiation (IR)‑induced damage [5]. However, there remains a dearth of comprehensive review on the role of pyroptosis in the antitumor effects induced by radiation. This review aims to offer a thorough and comprehensive overview of pyroptosis, encompassing its discovery, mechanism, and association with RT of cancer. Additionally, it primarily discusses how RT induces ICD, specifically pyroptosis, looking for breakthroughs in mechanisms and therapeutic approaches for RT combined with immunotherapy.

## Mechanism and features of pyroptosis

**Fig. 1:**
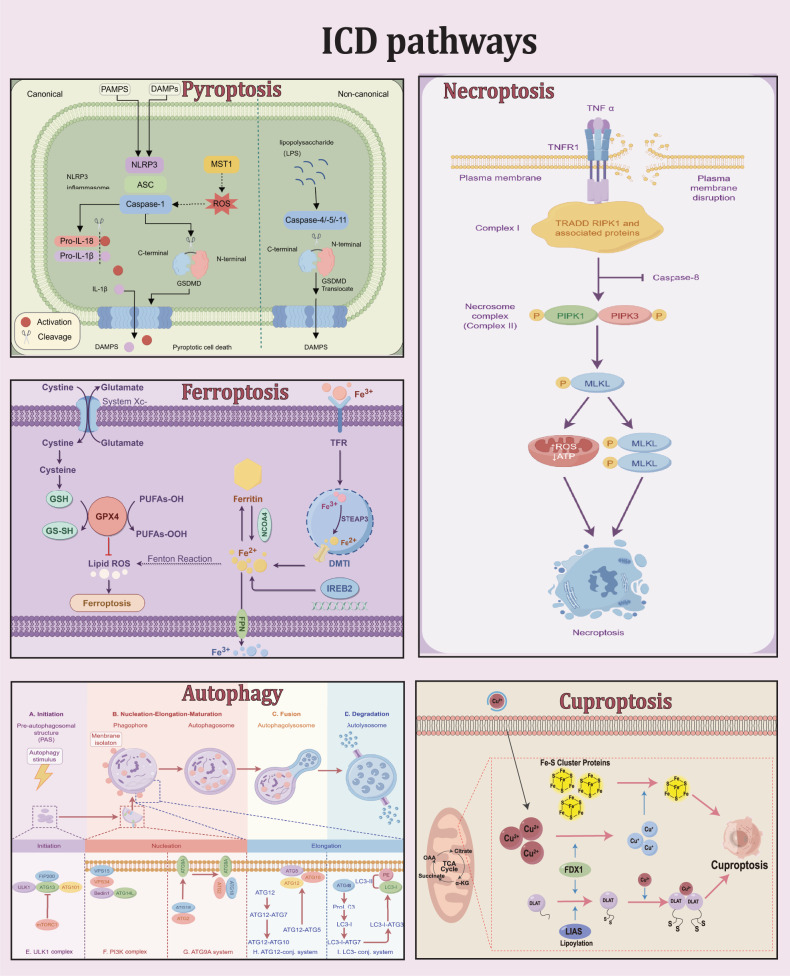
ICD mainly includes ferroptosis, cuproptosis, autophagy, necroptosis, and pyroptosis. The main feature of ferroptosis is lipid peroxidation, mainly due to the over-enrichment of iron-dependent lipid ROS and GPX4 clearance. The main mechanisms are divalent iron ions catalyze the Fenton reaction to produce lipid ROS, inducing cell death, and as an important marker of ferroptosis, GPX4 can be activated by GSH to protect cells from oxidative damage. The detailed mechanism of cuproptosis is unknown, but it has been established that copper reduces Fe-S cluster protein levels and induces cell death by targeting direct binding to lipoylated TCA cycle proteins, and the key factor in this process is FDX1, which not only regulates the lipoylation of DLAT but also reduces Cu2+ to Cu+, leading to inhibition of Fe-S cluster protein synthesis and inducing cell death. Cu2+ binds to lipatoacylated DLAT and induces heterodimerization of DLAT can also induce cell death. The main feature of autophagy is that autophagy-related genes regulate the process by which lysosomal degradation of cytoplasmic proteins and damaged organelles. There are four key processes involved: initiation, nucleation, fusion, and degradation. The main regulators are ULK 1 complex PI3K complex ATG9A system ATG 12 and LC3. Necrosis is often induced by extracellular stimuli, such as the binding of TNF superfamily proteins to the cell membrane, resulting in the activation of downstream RIP kinases, and phosphorylation after binding of RIPK1 and RIPK3 is a key link in necroptosis, which subsequently leads to phosphorylation of MLKL, induces oligomerization, translocations to the plasma membrane, and leads to membrane rupture. The main characteristics of pyroptosis are the rupture of cell membranes and the release of immunogenic molecules, and the main mechanisms are: canonical pathway is inflammasome-induced death of caspase-1/GSDMD pathway, non-canonical pathway is LPS-induced caspase-4/5/11-GSDMD pathway.

ICD represents a distinct form of Regulated cell death (RCD) that is initiated by stress stimuli and has the capacity to elicit adaptive immune responses targeting antigens derived from deceased cells [6], including pyroptosis, ferroptosis, necrotic apoptosis, among others (Fig. 1). Radiation-induced ICD is characterized by the emergence of DAMPs, thereby modulating the tumor microenvironment (TME) and promoting the propagation of anti-tumor immunity. Consequently, the role of RT extends beyond local lesion control, as it also possesses valuable immunomodulatory properties. Pyroptosis, an ICD triggered by radiation, has demonstrated tumor-suppressive effects and the ability to stimulate anti-tumor immune responses. The primary distinction between pyroptosis and other forms of ICD lies in its reliance on the activation of the caspase-1 pathway. Caspase-1, a cysteine protease, is accountable for the proteolytic cleavage and subsequent activation of pro-inflammatory cytokines, namely interleukin-1β (IL-1β) and interleukin-18 (IL-18). These pro-inflammatory cytokines assume a pivotal role in the immune response. For instance, the activation of IL-1 signaling within dendritic cells (DCs) was found to facilitate the development of radiation-induced antitumor immunity through the augmentation of DCs’ cross-priming capabilities [7]. Another difference is the role of the inflammasome in pyroptosis. The exposure to IR can incite the liberation of DAMPs from the expiring tumor cells, thereby activating the inflammasome and instigating pyroptosis. Consequently, this mechanism leads to the recruitment of immune cells to the tumor location and the provocation of an immune reaction against the irradiated neoplastic cells.

In 2001, Cookson and Brennan first introduced the notion of pyroptosis as a distinct form of cell death in inflammatory cells, characterized by its reliance on caspase-1, which is a completely different mode of death from apoptosis [8]. For a long time since the concept of pyroptosis was proposed, there has been no significant progress in its mechanism. It was not until 2015 that the key molecular mechanism of pyroptosis was elucidated for the first time by Shao Feng’s team [9]. They confirmed that GSDMD, a member of the gasdermin (GSDM) family, is a direct molecule of inflammatory caspases to induce pyroptosis, revealing for the first time that the N-terminal domain of GSDM family proteins has the function of punching holes in the membrane and thereby destroying the cell membrane [10].

### Caspase-1-mediated canonical pathways

**Fig. 2:**
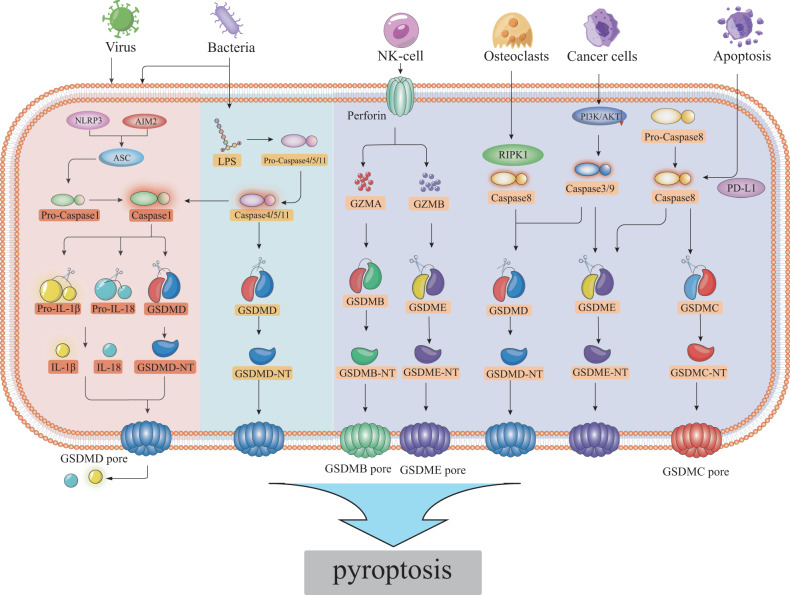
There are three ways in which pyroptosis occurs. (1) The caspase-1 pathway, with the inflammasome binding to pro-caspase-1 through the ASC adaptor protein. This activates caspase-1, which cleaves GSDMD into N-GSDMD and C-GSDMD terminals. Additionally, it activates pro-IL-1β and pro-IL-18. N-GSDMD binds to the cell membrane, forming membrane pores that release cellular contents. (2) The caspase-4/5/11 pathway, LPS-activated caspase-4/5/11 cleaves GSDMD to induce pyroptosis occurring; at the same time induces the activation of caspase-1. (3) Other pathways that cause pyroptosis, for example, caspase-3/8 cleavage GSDMD pathway, caspase-3/8/9 cleavage GSDME pathway, GZMA cleavage GSDMB pathway, GZMB cleavage GSDME pathway, and PD-L1 to convert apoptosis into caspase-8 cleavage GSDMC pathway in pyroptosis.

Caspase-1-mediated pathway of pyroptosis, also known as the inflammasome-mediated pathway (Fig. 2), canonical inflammasomes mainly include the NOD-like receptors (NLR) family, AIM2, and pyrin inflammasomes [11]. The inflammasome is a cytosolic protein complex that interacts with the adaptor protein ASC, resulting in the activation of caspase-1 [12]. Activated caspase-1 catalyzes the maturation of the pro-inflammatory cytokines IL-1β and IL-18. GSDMD, the critical protein that causes pyroptosis, is cleaved by caspase-1 into two parts: the C-terminal and N-terminal of GSDMD. The N-terminal structure binds to the cell membrane to form membrane pores, producing cell pyroptosis and releasing cell contents. In recent years, the activation of the cGAS-STING signaling pathway has been demonstrated to elevate the expression levels of NLRP3, caspase-1, and GSDMD, ultimately resulting in pyroptosis [13, 14].

### Caspase-4/5/11-mediated non-canonical pathways

Caspase-4/5/11-mediated non-canonical pathway, also known as the non-inflammasome mediated pathway (Fig. 2), activation of caspase-4/5 in humans and caspase-11 in mice is dependent on lipopolysaccharide (LPS), a component of the cell wall of Gram-negative bacteria, and does not require the involvement of the inflammasome [15]. Caspase-4/5/11 cleaves GSDMD, and the cleaved N-terminal structure accumulates on the cell membrane to produce pyroptosis. At the same time, caspase-4/5/11 activates caspase-1.

### Other pathways

Furthermore, several additional pathways have been discovered in recent years, in addition to the aforementioned pathways (Fig. 2). In the late stages of RANKL-induced osteoclasts, RIPK1, and caspase-8/3 can cleave GSDMD [16]. In lung cancer cells, inhibition of the PI3K/AKT signaling pathway can induce caspase-3 to cleave GSDME, causing pyroptosis [17]. Liu et al. study shows that the activation of caspase-3/8/9 induces GSDME-mediated pyroptosis [18]. Lymphocyte-derived granzyme A (GZMA) cleaves GSDMB, releasing membrane perforation activity of GSDMB [19]. Killer cell granzyme B (GZMB) activates target pyroptosis by cleaving GSDME directly at the same site as caspase-3 [20]. PD-L1 transforms TNFα-induced apoptosis into caspase-8-cleavage GSDMC and induces pyroptosis in cancer cells [21].

## Radiation regulates pyroptosis through various mechanisms

**Fig. 3:**
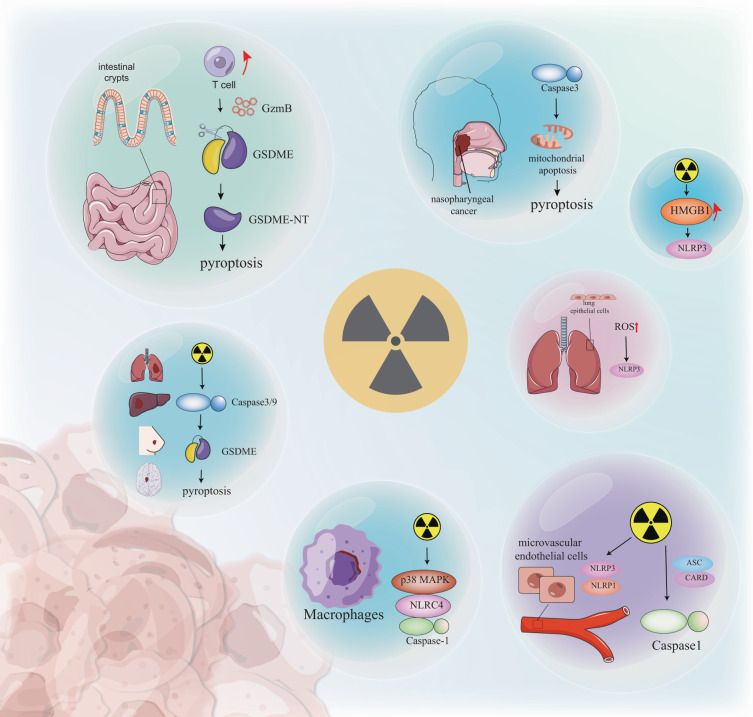
Several forms of RT-induced pyroptosis. γ radiation not only activates NLRP1 and NLRP3 complexes in microvascular endothelial cells, but also caspase-1. In lung epithelial cells, radiation-induced NLRP3 activation is associated with ROS accumulation. RT-induced HMGB1 activates the NLRP3 signaling pathway. Radiation activates p38 MAPK-NLRC4-caspase-1 in macrophages. Radiation induces pyroptosis in the caspase-9/caspase-3/GSDME pathway in a variety of tumors such as lung, liver, breast, and glioma. Excessive T cell responses in irradiated intestinal crypts further trigger GZMB-mediated GSDME lysis. In NPC cells, RT causes caspase-3-induced GSDME-dependent pyroptosis by the internal mitochondrial apoptosis pathway. Radiation can cause various manifestations of tumor pyroptosis and may trigger a variety of immune responses.

The modulation of pyroptosis can manifest through various mechanisms, encompassing epigenetic, transcriptional, and posttranslational processes. Here, we summarize the key molecular and cellular pathways that determine the vulnerability of cancer cells to pyroptosis. Furthermore, we aim to elucidate the specific impacts of IR on the occurrence and regulation of pyroptosis (Fig. 3).

Inflammasomes constitute an indispensable component in the initiation of the conventional pyroptosis pathway, and empirical investigations have demonstrated the capacity of RT to induce the activation of NLRP3 inflammasome [22]. γ radiation not only activates the NLRP1 and NLRP3 complexes in microvascular endothelial cells but also activates caspase-1 directly [23]. In lung epithelial cells, radiation-induced NLRP3 activation depends on glycolysis-associated reactive oxygen species (ROS) accumulation [24]. In addition, ROS involvement in radiation-induced NLRP3 activation has also been found in other cells [25], and irradiation-induced NLRP3 activation enhances lung hypersensitivity with more monocyte’s infiltration [25]. The presence of mitochondrial reactive oxygen species (mitoROS) leads to damage to oxidized mitochondrial DNA (mitoDNA), further activating NLRP3/caspase-1/GSDMD-dependent pyroptosis [26]. RT induces ICD, releasing DAMPs produced by dead cancer cells [27]. HMGB1, as a form of DAMPs, can be exposed to X-rays, increasing its expression and activating the NLRP3 signaling pathway [28]. The above studies have shown that radiation plays an important role in the activation of the inflammasome.

The occurrence of pyroptosis necessitates the presence of caspase-1, which plays a crucial role in cleaving GSDMD. Macrophages as abundant immune cells in TME, and radiation can activate p38 MAPK-NLRC4-caspase-1 in this cell [29]. Recent study has found that GSDME determines colorectal cancer (CRC) radiation sensitivity and radiation-related toxicity to surrounding normal tissue by caspase-3-dependent pyroptosis [30], the above-mentioned pyroptosis pathway recruits and activates natural killer cells to enhance anti-tumor immunity [30]. Radiation has been found to induce pyroptosis in the caspase-9/caspase-3/GSDME pathway in a variety of tumors such as lung, liver, breast, and glioma [31]. Meanwhile, BALB/c mice carrying GSDME overexpressing 4T1 tumors were found to promote anti-tumor immunity with enhanced tumor suppression, manifested by significantly elevated cytotoxic T lymphocyte levels and release of related cytokines [31]. In conventional irradiation doses, excessive T cell responses within irradiated intestinal crypts further trigger GZMB-mediated GSDME cleavage, producing more GSDME-N-terminal-driven pyroptosis than FLASH IR [32]. Intestinal pyroptosis as an ICD drives not only direct epithelial cell loss but also T cell recruitment to enhance local immune responses, thereby enhancing GSDME cleavage activity, and establishing T cell infiltration and pyroptosis [32]. In nasopharyngeal cancer (NPC) cells, RT causes caspase-3-induced GSDME-dependent pyroptosis via the internal mitochondrial apoptosis pathway [33], moreover, the upregulation of GSDME was found to enhance pyroptosis and radiosensitivity of NPC cells in vitro [33]. The aforementioned studies have demonstrated that radiation can elicit various manifestations of pyroptosis in tumors, encompassing cell membrane rupture, release of cellular contents, and activation of a robust inflammatory response. Furthermore, radiation has the potential to elicit a diverse array of immune responses within the tumor. Considering the significant involvement of GSDMD and GSDME in radiation-induced pyroptosis, we have compiled a comprehensive summary of their respective functions in RT, as presented in Table 1. Recent studies have also suggested that other members of the GSDMs family, such as GSDMA and GSDMB, may also be involved in pyroptosis and inflammation. However, the exact mechanisms by which these proteins contribute to pyroptosis are still being investigated.

**Table 1:** The role of GSDMD and GSDME in cancer RT.

GSDMs	Role	References
GSDMD	Radiation causes tissue damage by inflammasome-GSDMD signaling induced pyroptosis.	[49]
	GSDMD-induced pyroptosis can cause lung injury after radiation.	[64]
	The inflammasome-GSDMD axis causes a continuity of lethal radiation effects, initially affecting recipient cells and ultimately harming transplanted cells.	[49]
	Deficiencies of caspase-11 and GSDMD can significantly protect sepsis mice from radiation-induced injury and death.	[54]
	GSDMD-mediated pyroptosis is induced in mouse macrophages after irradiation.	[62]
	Microvascular endothelial cells undergo pyroptosis caused by GSDMD through gamma radiation.	[23]
GSDME	GSDME is a critical determinant of radiosensitivity in nasopharyngeal carcinoma.	[33]
	GSDME determines colorectal cancer radiosensitivity and radiation-related toxicity to surrounding normal tissues.	[30]
	GSDME-induced pyroptosis after radiotherapy enhances antigen presentation in DCs and promotes the infiltration of CD8 T cells.	[31]
	IR dose-dependently induces GSDME-mediated pyroptosis in HCT116 cells.	[68]

## Factors affecting RT-induced pyroptosis

IR can cause a variety of ICDs [34–38]. Previous research has extensively acknowledged ICDs as a mechanism governing cellular demise in reaction to antitumor treatment. In recent times, pyroptosis has emerged as a focal point of the investigation, with mounting evidence suggesting its potential anti-neoplastic effects in RT, chemotherapy, targeted therapy, and immunotherapy. Notably, a prior study revealed that pyroptosis accounted for 40–75% of nasopharyngeal carcinoma cell death induced by RT [33]. Consequently, further exploration of the underlying mechanisms of pyroptosis in the context of RT is warranted. There exist various factors that have the potential to influence the occurrence of pyroptosis induced by RT. The following factors are noteworthy for their potential impact on the induction of pyroptosis by RT:

### Radiation dose

The dose of radiation delivered to the tumor cells can affect the induction of pyroptosis. Higher radiation doses can trigger greater cellular stress and DNA damage [39]. DNA damage triggers AIM2-mediated and NLRP3-mediated pyroptosis [40, 41]. In addition, radiation quality can also affect pyroptosis. For example, studies have shown that high-linear energy transfer (LET) radiation, such as alpha particles, can induce more pyroptosis compared to low-LET radiation, such as X-rays or gamma rays.

### Tumor type

Different types of tumors may exhibit varying sensitivities to radiation-induced pyroptosis, which can be attributed to differences in their molecular and genetic makeup. For instance, non-small cell lung cancer (NSCLC) has been shown to have significantly increased levels of the GSDMD protein, which is associated with aggressive traits such as larger tumor size and advanced TNM staging [42]. Similarly, radiation-resistant lung adenocarcinoma (LUAD) cells have been found to have significantly upregulated levels of GSDMC [43]. In contrast, gastric cancer shows downregulation of GSDMA, GSDMC, and GSDMD proteins [44, 45]. Colorectal cancer (CRC) cells, on the other hand, have reduced GSDMD protein levels, which can inhibit the occurrence of CRC by activating GSDMD [46]. In the case of CRC, GSDME is silenced, but its expression has been found to make radiation-resistant CRC cells sensitive to radiation exposure [30]. These examples highlight how some tumors may be inherently more susceptible to pyroptosis due to their unique genetic and molecular characteristics, which in turn predispose them to cell death upon exposure to radiation.

### Immune system activation

Pyroptosis is associated with the activation of the immune system. Factors that enhance immune response, such as the presence of immune cells like macrophages and dendritic cells, can influence the extent of pyroptosis induction during RT. For example, IR can induce the production of NLRP3 in mouse bone marrow macrophages [5]. RT-activated NLRP3 can increase antigen presentation, innate function, and T cell priming, and promote radiation-induced immune priming and distant response in anti-PD1 resistance models [47]. Additionally, the activation of inflammasome pathways in splenocyte subsets increases as the systemic radiation dose increases [48].

### Inflammatory signaling pathways

Pyroptosis is regulated by specific signaling pathways, including the inflammasome pathway. High-dose radiation causes severe damage to bones and spleen through the mechanism of activating NLRP3 and AIM2 inflammasome while activating GSDMD [49], AIM2 inflammasome-mediated pyroptosis in macrophages and epithelial cells plays a key role in the development of radiation-induced tissue damage [40]. RT can activate NF-κB, a transcription factor that regulates the expression of genes related to inflammation and cell survival. NF-κB activation can promote the expression of pro-inflammatory cytokines and apoptosis-related genes, leading to pyroptosis [50]. The activation or inhibition of these pathways can impact the level of pyroptosis triggered by RT.

### Oxygen levels

Oxygen availability within TME can affect the efficacy of RT-induced pyroptosis. IR can produce ROS such as oxygen radicals and hydrogen peroxide through the radiation breakdown of cellular water and stimulation of oxidases [51]. Activation of ROS triggers caspase-8/GSDMC-induced pyroptosis, which can inhibit tumor growth and metastasis [52]. Radiation-generated reactive oxygen radicals activate caspase-9/3, causing GSDME-induced pyroptosis in tumor cells [31]. Hypoxic conditions may reduce the sensitivity of cancer cells to radiation-induced pyroptosis.

### Genetic factors

Variations in genes involved in pyroptosis signaling pathways may affect the response of cancer cells to RT-induced pyroptosis. The GSDM family of proteins has been demonstrated to exert a significant influence on the process of pyroptosis. More specifically, certain constituents of the GSDM family, including GSDMD and GSDME, undergo activation in response to inflammatory signals and subsequent cleavage by caspases. This enzymatic cleavage event results in the creation of pores within the cellular membrane, facilitating the release of pro-inflammatory cytokines. Consequently, the expression levels of GSDMs can exert a substantial impact on the occurrence of pyroptosis induced by RT.

Previous research has shown that the cGAS-STING pathway is significantly upregulated and NLRP3 is connected to the upstream DNA-sensing cGAS-STING pathway, inducing pyroptosis [53]. RT upregulated the caspase-11 pathway, while cGAS knockout significantly attenuated RT upregulated caspase-11 [54]. Genetic differences between individuals or tumor cells can influence the extent of pyroptosis induction.

### Treatment combinations

RT is often used in combination with other anti-cancer treatments like chemotherapy or immunotherapy. Genetic variants of CASP4, CASP11, GSDME, and NLRP3 are associated with the occurrence of adverse reactions in rectal cancer patients who receive chemoradiotherapy after surgery [55]. FLASH irradiation results in the inhibition of cascade feedback consisting of CD1 T cell chemotaxis and GSDME-mediated intestinal pyroptosis in the case of PD-L8 blockade [32]. The presence of concurrent treatments can modulate the effectiveness of pyroptosis induction during RT.

In recent times, there has been an identification of various pharmacological agents with the capacity to modulate pyroptosis. A comprehensive compilation of these agents has been presented in Table 2. These agents hold potential as candidates for modulating sensitizers in RT. Although the utilization of these candidates alongside RT is not commonly practiced in clinical settings, certain agents have exhibited promising clinical prospects. Notably, several pharmaceutical agents, including Vx-765, have demonstrated the ability to effectively inhibit pyroptosis and mitigate radiation-induced harm when combined with RT [50].

**Table 2:** Drugs that affect pyroptosis.

	Drugs	Mechanism	References
Inhibitors	Isoliquiritigenin	Attenuated SIRT3-mediated NLRP6-mediated pyroptosis in vascular endothelial cells.	[69]
	Oridonin analogs	Specifically targeted inhibition of the NLRP3 inflammasome.	[70]
	Necrosulfonamide	It reduces the expression of GSDMD, NLRP3, dissociation of caspase-1, caspase-11, and secretion of IL-1β.	[71, 72]
	Argon	Inhibition of NF-κB/NLRP3 inflammasome signaling pathway.	[73]
	Vx-765	It can inhibit caspase-1.	[74]
	Jujuboside A	It works by blocking inflammasome-mediated pyroptosis.	[75]
	Mcc950	It targets the NLRP3 ATP-hydrolysis motif for inflammasome inhibition.	[76, 77]
	Nicorandil	The AMPK/TXNIP/NLRP3 signaling pathway can inhibit pyroptosis.	[78]
	Ibrutinib	Inhibits pyroptosis-associated proteins (NLRP3, caspase-1, GSDMD, IL-1β and IL-18).	[79]
	RKIP	It can bind to ASC and then interrupt the assembly of NLRP3 inflammasomes.	[80]
	Tanshinone IIA sodium sulfonate	The AMPK signaling pathway inhibits vascular endothelial cell pyroptosis.	[81]
	Disulfiram	It can block GSDMD cleavage.	[82]
	Dectin-1	Dectin-1 blockade inhibits NLRP3 activation.	[83]
	Sennoside A	It inactivates caspase-1 to inhibit NLRP3 and AIM2 inflammasome-involved inflammation.	[84]
	Total extract of Abelmoschus manihot L	Inhibition of caspase-8/caspase-3/NLRP3/GSDME signaling.	[85]
	Dextmedetomidine	It inhibits GSDMD by activating the PI3K/AKT/GSK3β pathway.	[86]
	Esculetin	Inhibition of pyroptosis through NF-κB/NLRP3 signaling pathway.	[87]
Activators	Curcumin	It activates NLRC4, AIM2, and IFI16 inflammasomes and induces pyroptosis via upregulated ISG3 transcription factors in leukemic cells.	[88]
	Cathepsin B	Activation of NLRP3/caspase-1-induced pyroptosis.	[89, 90]
	Salsolinol	Activation of NLRP3-dependent pyroptosis.	[91]
	OTUD4	Induce pyroptosis by GSDME deubiquitination.	[33]
	Malotocin D	Resulting in cleavage of caspase-9 and caspase-3 induces GSDMD-associated pyroptosis.	[92]
	Silica Nanoparticles	It can activate the NLRP3 inflammasome.	[93]
	Gamboacid	It induces caspase-3-GSDME-dependent pyroptosis.	[94]
	Acrylamide	It induces the NLRP3-caspase-1-GSDMD-IL-1β/IL-18 pathway and the caspase-3-GSDME-IL-1β/IL-18 pathway.	[95]
	Caveolin-1	Pyroptosis is mediated by activation of ROS-dependent NLRP3 inflammasomes.	[96]
	Nitrosamines	It mediates the activation of NLRP3 by mitochondrial ROS.	[97]
	Nitidine chloride	Caspase-3/GSDME-dependent pyroptosis is induced by inhibiting the PI3K/Akt pathway in lung cancer.	[17]
	Luteolin	Increased expression of caspase-1, GSDMD and IL-1β.	[98]
	Myricetin	Activation of caspase-3-GSDME-induced pyroptosis by the ER stress pathway.	[99]
	Triclabendazole	Activates caspase-3/GSDME to induce pyroptosis in breast cancer cells.	[100]
	Secoisolariciresinol diglucoside	It can activate the caspase-1/GSDMD pathway.	[101]
	Bexarotene	Activates caspase-4 and GSDME.	[102]
	Neobractatin	Trigger GSDME-mediated pyroptosis in esophageal cancer cells.	[103]
	Germacrone	It promotes cleavage of caspase-3 and GSDME.	[104]
	Tricalcium phosphate particles	It promotes pyroptosis through ROS/NLRP3/caspase-1 pathway.	[105]
	Clostridium septicum α-toxin	Activation of NLRP3 by engaging GPI-anchored proteins.	[106]

## Potential therapeutic application of RT-induced pyroptosis

As an ICD, pyroptosis affects not only the tumor cells themselves but also the surrounding tumor immune microenvironment in tumors. Recent investigations have shed light on the involvement of pyroptosis in the context of cancer RT. One potential therapeutic application of RT-induced pyroptosis is in combination with immunotherapy. Immunotherapy aims to boost the body’s immune system to recognize and attack cancer cells. By combining RT-induced pyroptosis with immunotherapy, researchers hope to enhance the immune response against cancer cells, potentially improving treatment outcomes. Studies have found that RT-induced pyroptosis of tumor cells can stimulate the maturation and activation of bone marrow-derived dendritic cells and enhance antigen presentation. It further promotes CD8^+^ T cell activation and infiltration, and further kills tumor cells by releasing cytokines such as TNF-α and IFN-γ [31].

Moreover, RT-induced pyroptosis may have implications for overcoming tumor resistance to RT. The presence of resistance to RT has the potential to hinder the efficacy of treatment, making it a crucial aspect of concern in clinical investigations related to RT. Currently, the underlying mechanisms responsible for the development of radioresistance remain inadequately understood, but by inducing pyroptosis, it may be possible to overcome this resistance and improve tumor control. Recent studies have shown that Apyrimidinic Endodeoxyribonuclease 1 promotes the radioresistance of LUAD cells by targeting the STING signaling pathway and protects them from radiation-induced pyroptosis [56]. In esophageal cancer cells, sulconazole inhibits glycolysis and triggers enhanced mitochondrial oxidative stress, leading to PANoptosis, thereby enhancing RT sensitivity. PANoptosis is caused by the components of pyroptosis, apoptosis, and necroptosis [57]. miR-1290/NLRP3-mediated pyroptosis may play an important role in triple-negative breast cancer radioresistance [58]. The above studies have revealed the correlation between radioresistance and pyroptosis in some aspects, which to a certain extent prevents the radioresistance and enhances the sensitivity of RT.

Acute radiation lung injury (ARILI) is a serious complication caused by RT to the chest [59]. The main causes of ARILI are immune and inflammatory dysfunction [60, 61]. miR-223-3p can improve radiation-induced macrophage pyroptosis and lung damage [62]. Macrophages play an important role in acute lung injury [63]. ACT001 ameliorates radiation-induced lung injury by inhibiting NLRP3-induced pyroptosis [64]. Artemisia annua in Re-Du-Ning injection inhibits radiation-triggered AIM2-induced pyroptosis, thereby alleviating radiation-induced pneumonia [65]. Radiation-induced enteropathy (RIE) is also one of the most common complications, and there are currently no effective interventions. Studies have found that micheliolide-mediated autophagy improves RIE by inhibiting NLRP3 degradation [66]. p-coumaric acid improves radiation-induced intestinal damage by inhibiting oxidative stress, inflammatory response, and pyroptosis [67]. These studies have revealed that pyroptosis plays an important role in RT injury. However, it’s important to note that the field of RT-induced pyroptosis is still under investigation, and more research is needed to fully understand its therapeutic potential. Clinical trials and further studies will provide a better understanding of how to harness this process for cancer treatment.

## Concluding remarks

Pyroptosis is a form of ICD that plays a crucial role in the immune response against pathogens. In recent years, researchers have discovered its potential therapeutic applications in cancer RT. During RT pyroptosis could lead to the release of immune factors such as IL-18 and IL-1β, which in turn facilitate the promotion of anti-tumor immunity. Additionally, pyroptosis induced by IR plays a crucial role in tumor suppression by initiating the activation of anti-tumor immune responses [30, 31]. One of the key biological functions of pyroptosis is its ability to trigger inflammation and stimulate the release of pro-inflammatory cytokines. By inducing pyroptosis in cancer cells, it can activate the immune system and enhance anti-tumor immune responses. Pyroptosis also eliminates damaged cells and prevents the accumulation of genetic alterations that can lead to the development of cancer. This mechanism is particularly important in RT, as it helps to eliminate cancer cells that survive radiation treatment and reduce the risk of tumor recurrence. Furthermore, as an ICD, it could promote the release of tumor-associated antigens and enhancing the recognition of cancer cells by the immune system. This immune recognition can potentially lead to the development of long-lasting anti-tumor immune responses, improving the overall effectiveness of cancer treatments.

In terms of therapeutic potentials, studies are exploring various strategies to induce pyroptosis in cancer cells. This includes the use of specific drugs or genetic modifications to enhance the sensitivity of cancer cells to pyroptosis-inducing signals. Additionally, combination therapies involving radiation therapy and agents that promote pyroptosis are being investigated for their potential synergistic effects in cancer treatment.

Overall, understanding the biological functions of pyroptosis and its therapeutic potentials in cancer RT holds promise for the development of novel treatment strategies that can improve patient outcomes. However, further research and clinical trials are needed to fully explore and validate these potentials.
